# Parentification, distress, and relationship with parents as factors shaping the relationship between adult siblings and their brother/sister with disabilities

**DOI:** 10.3389/fpsyt.2022.1079608

**Published:** 2023-01-18

**Authors:** Annalisa Levante, Chiara Martis, Cristina Maria Del Prete, Paola Martino, Ferruccio Pascali, Patrizia Primiceri, Massimo Vergari, Flavia Lecciso

**Affiliations:** ^1^Department of Human and Social Sciences, University of Salento, Lecce, Italy; ^2^Lab of Applied Psychology, Department of Human and Social Sciences, University of Salento, Lecce, Italy; ^3^District for Rehabilitation, Local Health Service, Lecce, Italy; ^4^Office for Inclusion of People with Disabilities, University of Salento, Lecce, Italy

**Keywords:** sibling, disability, parentification, distress, sibling relationship, sibling-parents relationship, serial mediation model

## Abstract

According to parentification theory, when the siblings of a brother/sister with disabilities assume parent-like duties, this role reversal is known as sibling-focused parentification. It has a significant impact on these siblings’ distress and the quality of their family relationships; 605 Italian adult siblings (19–26 years) of people with disabilities completed the online survey. Measures of siblings’ parentification, distress, quality of family relationships, social support, and perceived benefits of parentification were used. The hypothesized model aims to test, on the target sample, the distress and the quality of the relationship with parents as mediators on the interplay between the siblings’ parentification and their sibling relationship. Additionally, social support and perceived benefits of parentification as protective factors were considered. Results showed that the distress and the low quality of the relationship with parents negatively affected the interplay between the siblings’ parentification and the relationship with their own brother/sister with disabilities. Social support and the perceived benefits of parentification decreased the siblings’ distress levels; the perceived benefits of parentification served as a protective factor for the quality of the relationship with parents. Current findings extend the knowledge regarding the risk and protective factors of the siblings’ mental health when disability occurs in the family. Additionally, they inform family-based intervention programs, which should involve the whole family system for reducing distress and improving the wellbeing of siblings without disabilities.

## 1. Introduction

Family systems theory states that all family members influence each other (1, 2). When an individual with disabilities is present in the family, examining its impact on the psychological functioning of each member is critical. The majority of the studies involving families with a child with disabilities devoted attention to mothers because they are usually the main caregiver. To be specific, mothers of children with disabilities showed high levels of stress and depression (3–5), social isolation (6), distress (7, 8), and difficulties with job-related activities (9, 10). In addition, a growing amount of evidence involved fathers (11, 12), highlighting that they showed fewer depression symptoms than mothers and a high over-investment in work activities (13). Furthermore, in the past decades, research investigations devoted attention to the psychological impact of the disabilities of a brother/sister on the sibling(s) without disability. Findings regarding this topic were mixed. Some studies (14–16) reported a negative impact of disabilities on the psychological functioning of siblings without disabilities, in terms of feelings of rejection toward the brother/sister with disabilities, guilt, anxiety and depression symptoms, lower levels of wellbeing, and aggressive behaviors, whereas other studies (17, 18) found a positive influence in terms of increased levels of responsibility, high levels of self-efficacy and cognitive/emotional empathy, high tolerance and responsiveness levels, as well as positive feelings about caring for their brother/sister with disabilities. Finally, a few studies (19, 20) found no differences in terms of functioning between siblings of people with disabilities and siblings of typically developing ones.

Among the psychological factors examined in literature on the siblings of people with disabilities population, the frequently investigated factors are (a) sibling-focused parentification (21, 22), which is the parent-like role performed by the siblings toward the brother/sister with disabilities (22); (b) sibling relationship (23, 24) in terms of both positive (e.g., empathy) and negative (e.g., distress) aspects of the relationship resulting from growing up with a brother/sister with disabilities; and (c) the emotional adjustment (14, 25) in terms of the emotional response resulting from the challenges related to caregiving of the brother/sister with disabilities (26).

In the current article, we focused both on these psychological factors and the interplay between them. To be specific, we paid attention to the impact of sibling-focused parentification on (a) the relationship between study participants and their brother/sister with disabilities, (b) the relationship between study participants and their parents, and (c) the siblings’ emotional adjustment, in terms of distress levels. To investigate these relationships, we hypothesized a serial mediation model that engulfed and tested them.

### 1.1. Sibling-focused Parentification, relationship with siblings and with Parents, and emotional adjustment

When an individual assumes parent-like duties toward a family member, this role reversal is known as parentification (16). According to Hooper et al. (16, 22), this family dynamic consists of three factors: (1) The first one is parent-focused parentification: it captures the roles and responsibilities that the child or adolescent without disabilities addresses toward their own parent(s); (2) the second factor is the sibling-focused parentification: it captures the roles and responsibilities toward the brother/sister; and (3) the third factor is the perceived benefits of parentification, which consists of positive states of mind (i.e., emotions and thoughts) associated with caregiving roles and responsibilities served in the family because of the parentification. Among these three factors of parentification, according to the specific study aims, we focused our attention only on sibling-focused parentification (as a risk factor) and the perceived benefits of parentification (as a protective factor).

Overall, evidence showed that sibling-focused parentification plays a pivotal role in the sibling relationship. This relationship was examined in literature (17, 18, 23, 27–35), and mixed findings were reached. To be specific, on the one hand, some studies (27–29) highlighted that sibling-focused parentification was associated with a positive sibling relationship; in other words, the time spent in caregiving led to a warm, close, and satisfied relationship between siblings. Again, several studies (17, 18, 30) found positive outcomes of parentification on the functioning of siblings of people with disabilities, in terms of increased responsibility, empathy, and maturity. On the other hand, detrimental outcomes because of parentification were found: in particular, high levels of perceived responsibility (31, 32) and low quality of life (33) were found. In addition, the shame experienced by siblings of people with disabilities because of their brother/sister with disabilities (23), the growing concerns because of their future caregiver’s role (34), and the work and social difficulties of siblings without disabilities because of the parentification (35) negatively affected the sibling relationship.

In families where one of the children is a person with disabilities, sibling-focused parentification is a process that not only impacts the relationship between siblings but also indirectly influences the relationship between the child who is not the person with disabilities and the parents (henceforth relationship with parents). Some studies (36, 37) found that this relationship was characterized by conflict related to a brother/sister with disabilities. In particular, studies (17, 38) reported that siblings without disabilities experienced a lack of communication with parents regarding the brother/sister with disabilities which, in turn, led them to conceive the caregiving as forced and an obvious requirement. In addition, the quality of the relationship with parents was characterized by grudges because more attention and care were addressed to brothers/sisters with disabilities (38). This results in siblings’ reactions of jealousy, anger, and grudge, which, in turn, negatively affects the relationship with their brother/sister with disabilities (17, 38).

Finally, sibling-focused parentification also affects the siblings’ emotional adjustment. Evidence (14, 15) showed that sibling-focused parentification was related to siblings’ negative emotional adjustment [e.g., feelings of rejection, guilt (39)], social withdrawal, and psychopathology (e.g., anxiety disorders, personality disorders, and eating disorders). In addition, a meta-analysis (16) found that the parentification experienced in childhood was associated with psychopathology in adulthood (in terms of greater levels of anxiety and depression symptoms, and global distress). To the best of our knowledge, there was a paucity of studies focused on the interplay between sibling-focused parentification and the emotional adjustment–in terms of distress–of the siblings of people with disabilities.

**FIGURE 1:**
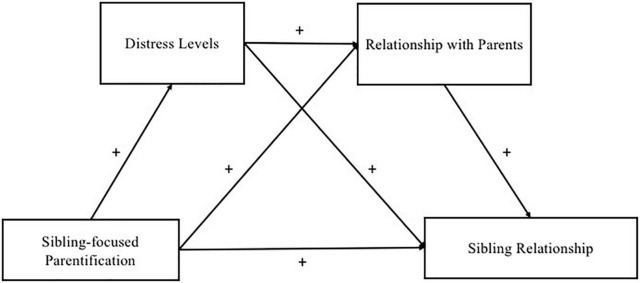
The hypothesized serial mediation model.

With this theoretical background in mind and due to the longevity of the sibling relationship, studies examining the psychological risk and protective factors that affect sibling relationships are critical. A serial mediation model was conceived: the sibling-focused parentification is the independent variable (X), the sibling relationship is the predictor (Y), and the distress of the sibling without disabilities (M1) and the low quality of the relationship with parents (M2) served as mediators. With regard to the relationships with brother/sister with disabilities and with parents, we conceived them as consisting of negative aspects (e.g., guilt, too much responsibility, misunderstanding, and indifference) that may affect the relationships. In addition, in our serial mediation model, we examined the effect of two protective factors that could help the siblings of people with disabilities to cope with stressors they encounter in caregiving a brother/sister with disabilities: the first factor was the social support (39, 40) and the second one was the perceived benefits of parentification (27, 41). Figure 1 shows the hypothesized serial mediation model, the direction, and the nature (positive vs. negative) of the relationship.

## 2. Materials and methods

### 2.1. Study design

The study has been carried out in Italy between April and June 2021 *via* an online survey imported on Microsoft Forms and disseminated *via* the main social platforms (e.g., WhatsApp and Facebook), and *via* the mailing list of non-profit associations affiliated with local health services (i.e., ASL Lecce) (e.g., “We can” association; Psifia association) applying the snowball sampling strategy for data collection. The predefined study sample inclusion criteria were (1) having a brother/sister with any disabilities and (2) being aged between 19 and 26 years. The exclusion criterion was the presence of any disability for the person participating in the survey.

Two theoretical models were considered to establish the participants’ age range choice. First, according to Havighurst’s theory of developmental tasks (42), individuals aged 19–26 years are young adults who have specific developmental tasks to achieve, i.e., autonomy, defining their own identity, building long-term social relationships, and establishing a professional career. Second, in accordance with Arnett (43), this age range was included in emerging adulthood during which the individuals developed their own identity. The caregiver role assumed by siblings of people with disabilities might involve excessive investment within the family system hindering the accomplishment of some of these development tasks (42) as well as the development of adult identity (43). Prior to the study, the University Ethical Committee of the Department of Human and Social Sciences of the University of Salento approved the research (No. 0056300; 25/03/2021) and study participants approved the e-consent form before their participation.

### 2.2. Participants

The questionnaire was filled out by 605 adult siblings of people with disabilities aged 19–26 years [M (SD) = 22.49 (2.91) years]. The majority of the participants were women (*n* = 570; 94.2%), and more than half of the participants (57.5%) were older than their brother/sister with disabilities. Among the study participants, 50.5% lived in Southern Italy, 28.26% lived in Northern Italy, and 21.24% lived in Central Italy. Based on the educational levels, the study participants were divided into three levels: 2.1% of participants were found to have a low educational level (up to 13 years of education), 3.5% of participants were found to have an intermediate educational level (up to 16 years of education), and 94.4% of participants were found to have a high educational level (18 or more years of education); 477 (78.9%) participants were single and 128 (21.1%) participants were in a relationship.

With regard to the brother/sister with disabilities, 53.1% of them were females [M (SD) = 25.08 (9.08) years]. We asked the participants to report the disability of his/her brother/sister, and, in accordance with expert clinicians, five types of disabilities were defined: (1) genetic diseases (9.8%), (2) physical disorders (24.6%), (3) multiple disabilities (19.7%), (4) neuropsychiatric disorders (42.3%), and (5) sensory disabilities (3.6%).

### 2.3. Measures

#### 2.3.1. Information regarding disabilities

Additional information regarding the disabilities of the brother/sister was collected using the following *ad doc* questions: (i) *How did you know about your brother/sister’s disabilities?*; (ii) *How often each family member is caregiving for the brother/sister with disabilities?*; (iii) *How much did the brother/sister’s disabilities impact on your everyday life?*; and (iv) *How often you referred to the professionals who care the brother/sister with disabilities?*

For question (ii), the response options vary from 1 (never) to 4 (always); for question (iii), the response options vary from 1 (not at all) to 4 (a lot); finally, for question (iv) the response options vary from 1 (never) to 5 (always).

#### 2.3.2. Siblings-focused parentification and perceived benefits of parentification

To collect data regarding siblings-focused parentification and the perceived benefits of parentification, two subscales of the self-report questionnaire Parentification Inventory [PI; (22)] were administered. To be specific, we used only the items regarding sibling-focused parentification (SFP) and the perceived benefits of parentification (PBP) subscales. SFP captures parent-like roles assumed by the siblings and the burden of responsibilities related to care for their brother/sister [M (SD) = 2.38 (0.85); α = 0.83; *r* > 0.317]; the second subscale (PBP) evaluates positive states of mind (e.g., feelings of appreciation from the family members and perceiving the family as a team) related to parentification [M (SD) = 3.48 (1.05); α = 0.86; *r* > 0.659]. For the purpose of the present study, we excluded the items regarding parents-focused parentification.

Two study’s authors translated the English version of the two administered sub-scales items into Italian. Furthermore, in our online survey, we edited the instructions, and we asked the participants to refer to their own brother/sister with disabilities. Response options vary from 1 (never true) to 5 (always true), and the two subscales were calculated as the mean of items according to Hooper et al. (22), with higher scores indicating higher levels of responsibility related to the parentification (sibling-focused parentification subscale) and more perceived benefits of parentification.

#### 2.3.3. Siblings’ distress

The levels of participants’ distress were measured by administering the Depression Anxiety Stress Scale-21 (DASS-21) self-report questionnaire (44). As suggested by Bottesi et al. (44), distress is a general psychological trait of adults characterized by a combination of depression symptoms, anxious traits, and stress symptoms. Response options varied from 0 (never happened to me) to 3 (always happened to me). The final score was calculated as the mean of all the items, with higher scores indicating higher distress levels [M (SD) = 2.06 (0.57); α = 0.94; *r* > 0.343].

#### 2.3.4. Negative aspects of sibling relationship and relationship between the sibling of people with disabilities and parents

A set of *ad hoc* questions were created in order to evaluate the negative aspects of the relationship between the siblings without disabilities and both parents (eight items) as well as between these young adults without disabilities and their brother/sister with disabilities (eight items). To be specific, the negative aspects explored were shame, guilt, too much responsibility, too much protection, misunderstanding, social withdrawal, aggressive behaviors, and indifference. We asked the study participants to answer how much each aspect characterized the relationships with both parents and with brother/sister with disability separately. Response options vary from 1 (not at all) to 4 (a lot). Two final scores were calculated as the mean of all eight items, with higher scores indicating a more negative relationship with brother/sister with disability [M (SD) = 1.98 (0.51); α = 0.71; *r* > 0.449] and with parents [M (SD) = 2.01 (0.53); α = 0.74; *r* > 0.431].

#### 2.3.5. Perceived social support

The Multidimensional Scale of Social Perceived Support [MSPSS; (45)] questionnaire was administered to evaluate the social support perceived by the study participants, including the support they get from family, friends, and significant others. Response options varied from 8 (very strongly disagree) to 7 (very strongly agree). The total score was calculated as the mean of all 12 items, with high scores indicating high social support [M (SD) = 5.46 (1.13); α = 0.90; *r* > 0.378].

#### 2.3.6. Covariates

The total score of the perceived social support, the perceived benefits of parentification subscale, the gender of the study participants and their brother/sister with disabilities, and five types of disabilities were included as covariates in the serial mediation model.

### 2.4. Data analysis plan

In this study, SPSS version 25 (46) was used. No missing data imputation techniques were computed because all administered items required a mandatory answer. Preliminary comparisons between psychological factors were performed: Mann-Whitney *U*-tests according to the gender of the study participants and the brother/sister with disabilities; and Kruskal-Wallis *H*-tests for five types of disabilities of the brother/sister. Pearson’s *r* correlations were computed. The serial mediation model was performed using Process v3.0 (5,000 bootstraps). In the serial mediation model, the predictor variable was the sibling-focused parentification (x), the outcome was the sibling relationship (y), and the mediators were the distress of the siblings without disabilities (M1) and the relationship with parents (M2). The social support, the perceived benefits of parentification, the gender of the siblings without disability and of the brother/sister with disabilities, and the five types of disabilities were included as covariates.

## 3. Results

### 3.1. Preliminary comparisons

Frequencies and descriptive statistics regarding the information on disabilities as reported by siblings are tabulated in Table 1.

**TABLE 1:** Frequencies and descriptive statistics regarding the information on the disabilities of the siblings of the participants.

	[n (%)]
***How did you know about your brother/sister’ disabilities?***	
By parents	292 (48.3%)
By myself	227 (37.5%)
By others	3 (0.5%)
Not remember	83 (13.7%)
(ii) *How often each family members are caregiving for the brother/sister with disabilities?*	*[M (SD); range 1 (never)–4 (always)]*
Mother	4.76 (0.61)
Father	4.11 (1.11)
Both parents	4.3 (1.08)
Older brothers/sisters	3.43 (1.29)
Younger brothers/sisters	3.33 (1.32)
Other (e.g., babysitter; grandparents)	2.73 (1.31)
(iii) *How much did the brother/sister’ disabilities impact on your everyday life?*	*[M (SD); range 1 (not at all)–4 (a lot)]*
Lifestyle choice	2.5 (1.02)
Concerns about the own future	2.49 (1.09)
Leaving the family house	2.48 (1.12)
Reflect on own caregiver role	3.15 (0.88)
Having a romantic relationship	2.37 (1.11)
Study or work	2.24 (1.14)
(iv) *How often you referred to the professionals who care the brother/sister with disabilities?*	*[M (SD); range 1 (never)–5 (always)]*
General practitioners	2.82 (1.23)
Expert practitioners	2.72 (1.29)
Physiotherapists	2.13 (1.37)
Speech therapists	2.33 (1.36)
Educators	2.72 (1.44)
Occupational therapist	1.84 (1.23)
Psychologists	2.05 (1.21)
Social workers	1.72 (1.14)
Special teachers	2.87 (1.45)

Regarding the participants’ gender, results showed significant differences in depression (*U* = 8,029; *p* = 0.05) and distress (*U* = 7,886; *p* = 0.037) levels; in particular, women showed higher levels of depression symptoms [M (SD) = 14.36 (5.36)] and distress [M (SD) = 42.77 (13.1)] than men [depression symptoms: M (SD) = 12.83 (5.46); distress: M (SD) = 38.6 (13.15)].

No differences in considered variables were found according to the gender of brother/sister with disabilities. The pairwise comparison between the five types of brother/sister disabilities showed a significant difference for the Perceived Benefit of Parentification subscale: findings showed a significant difference between siblings of people with physical disorders and multiple disabilities (*H* = 60.476; *p* = 0.047). Furthermore, results reported a significant difference between siblings of people with neuropsychiatric disorders and those with physical disorders (*H* = 52.729; *p* = 0.033). To be specific, siblings of brothers/sisters with physical disorders reported higher [M (SD) = 3.7 (1)] perceived benefits of parentification than siblings of people with multiple disabilities [M (SD) = 3.34 (1.08)] and with neuropsychiatric disorders [M (SD) = 3.38 (1.03)].

### 3.2. Correlations analyses

The results of correlations between the considered study variables are reported in Table 2. In sum, results showed that sibling-focused parentification was positively associated with the relationship with siblings, relationship between study participants and their parents, and siblings’ distress; in other words, the higher the sibling-focused parentification, the lower the quality of the relationship with the parents and relationship with the sibling with disabilities. Moreover, the more sibling-focused parentification the participants experienced, the higher distress levels they reported.

**TABLE 2:** Correlation between study variables.

Psychological factors	Perceived benefits of parentification	Sibling relationship	Relationship with parents	Distress	Social support
Sibling-focused parentification	−0.105***	0.183***	0.216***	0.301***	−0.153***
Perceived benefits of parentification		−0.352***	−0.569***	−0.422***	0.634***
Sibling relationship			0.632***	0.373***	−0.259***
Relationship with parents				457***	−0.444***
Distress					−0.425***
Social support					

*p < 0.05; **p < 0.01; ***p < 0.001.

Furthermore, findings showed that sibling-focused parentification was negatively associated with perceived benefits of parentification and social support: the more sibling-focused parentification the study participants experienced, the fewer benefits of parentification and social support they perceived.

### 3.3. Serial mediation model

**FIGURE 2:**
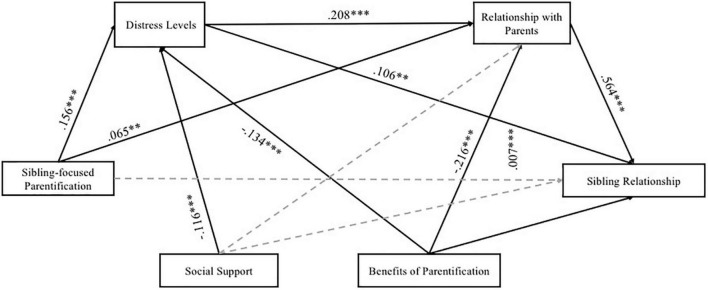
Serial mediation model testing the expected mediation effects. *p < 0.05; **p < 0.01; ***p < 0.001. Non-significant paths were displayed by dotted lines.

The serial mediation model results are reported in Table 3 and Figure 2. The serial mediation model was significant [*F* (7, 597) = 33.450; *p* < 0.001]. The total effect was significant (β = 0.091; SE = 0.023; *p* < 0.001). The direct path between sibling-focused parentification and sibling relationship was not significant (β = 0.020), whereas the paths between sibling-focused parentification and siblings’ distress (β = 0.156) and relationship with parents (β = 0.065) were significant. In other words, results indicate that more parentification toward the brother/sister with disabilities could have led the siblings without disabilities to perceive high distress levels and to experience more negative aspects in the relationship between study participants and parents. Furthermore, the paths between the distress and sibling relationship (β = 0.106) and relationship with their parents (β = 0.208) were significant, indicating that high levels of distress might have led the siblings of people with disabilities to experience more negative aspects in the relationships with brother/sister with disabilities and parents. The path between the relationship with parents and the sibling relationship was significant (β = 0.564); in other words, the low quality of the relationship with parents could have led to experiencing more negative aspects in the sibling relationship. Three indirect effects were found (refer to Table 3). The first one was regarding the mediating role of distress: sibling-focused parentification was significantly associated with distress, which, in turn, was significantly associated with the sibling relationship. The second mediating effect was related to the role of the relationship with parents: again, sibling-focused parentification was significantly associated with the relationship with parents, which, in turn, was significantly associated with the sibling relationship. Finally, the third indirect effect was related to the role served by the distress and the relationship with parents on the relationship between the sibling-focused parentification and the sibling relationship: the sibling-focused parentification was significantly associated with the distress and the relationship with parents, which, in turn, was significantly associated with the sibling relationship.

**TABLE 3:** Betas coefficients, standard errors, *p*-values, and bootstrap confidence intervals of serial mediation model.

Path		β	SE	*p*	95% Bootstrap CI	
					BootLLCI	BootULCI
Sibling-focused parentification →	Siblings’ distress	0.156	0.024	< 0.001	0.109	0.202
	Relationship with parents	0.065	0.021	0.002	0.022	0.108
	Sibling relationship	0.020	0.020	0.326	−0.021	0.058
Siblings’ distress →	Relationship with parents	0.208	0.035	< 0.001	0.137	0.279
	Sibling relationship	0.106	0.034	0.002	0.036	0.180
Relationship with parents →	Sibling relationship	0.564	0.039	< 0.001	0.477	0.650
**Indirect paths**						
Sibling-focused parentification → Siblings’ distress → Sibling relationship		0.028	0.010	-	0.009	0.049
Sibling-focused parentification → Relationship with parents → Sibling relationship		0.062	0.021	-	0.022	0.103
Sibling-focused parentification → Sibling distress → Relationship with parents → Sibling relationship		0.031	0.008	-	0.017	0.047

With regard to the impact of the covariates on the mediators and the outcome, findings showed that social support negatively impacted the distress: the lower the level of social support perceived by the siblings of people with disabilities, the higher the level of their distress, whereas the impact of social support on the relationship with parents (M2) and the sibling relationship (outcome) was not significant. The perceived benefits of parentification impacted both the mediators, indicating that experiencing positive states of mind because the parentification could lead siblings to perceive low distress levels and low quality of the relationship with parents. The impact of the perceived benefits of parentification on the sibling relationship was not significant. Finally, no significant impacts of the gender of the study participants and the brother/sister with disabilities, and five types of disabilities were found.

## 4. Discussion

The novel issue of the current article was to consider simultaneously the quality of the relationships between the siblings of people with disabilities and their parents as well as with their siblings with disabilities. The present study aimed to build a complex model exploring the impact of sibling-focused parentification on the sibling relationship *via* the serial mediation role of the siblings’ distress and the quality of the relationship with parents. Although this study is the first application of this hypothesized serial mediation model on the Italian siblings’ population, we believe that it could extend the knowledge regarding the topic and inform family-based intervention programs. These intervention programs could involve the entire family system, promoting wellness according to the Complete Mental Health (CMH) model by Keyes (47).

Regarding our findings, the preliminary analyses regarded the comparison across gender of siblings and brother/sister with disabilities on the considered psychological factors, and they were supported by previous studies; they should be interpreted cautiously because in our sample gender was not balanced.

In addition, we compared psychological factors (i.e., parentification, distress, relationship with parents, sibling relationship, social support, and perceived benefits of parentification) among people who are siblings of people with five different types of disabilities. Findings showed that siblings of brothers/sisters with physical disorders perceived more benefits of parentification than siblings of those with neuropsychiatric disorders and multiple disabilities. These findings could be explained by devoting attention to the care and management that the specific type of disability requires. For both groups, caregiving met severe difficulties because of the complex and pervasive nature of the neuropsychiatric disorders and the multiple disabilities (48–52). These reasons could lead the siblings to not perceive many benefits of parentification.

Regarding the main study purpose, that is, testing the serial mediation model, our results supported previous studies that found the dyadic relationship between sibling-focused parentification and distress levels (27, 39) as well as low quality relationships (38). Regarding the relationship between the distress of siblings without disabilities, the low quality of relationship with parents, and the sibling relationship, there is a paucity of studies (38, 53). Furthermore, using the tested serial mediation model, it was noteworthy that the distress levels and the quality of the relationship with parents mediated the relation between sibling-focused parentification and sibling relationship. Specifically, results showed that sibling-focused parentification, in terms of assuming parent-like roles and high responsibility toward the brother/sister, could predict high distress levels (17, 27) and low quality of the relationship with parents (38). In turn, these two mediators (i.e., distress and quality of relationship with parents) could have an effect on the sibling relationship; in other words, high distress levels and low quality of relationship with parents could negatively affect the sibling relationship, as found by others (53, 54). Furthermore, high distress levels might lead siblings without disabilities to perceive a more low quality relationship with their parents (38). All significant paths tested in our serial mediation model were in line with previous studies (31, 32) and showed a sharp vicious circle that started from the sibling-focused parentification and impacted both the distress levels of the siblings of people with disabilities and their general wellbeing (in terms of quality of family relationships).

Furthermore, our serial mediation model tested the role played by social support and the perceived benefits of parentification as protective factors, which help the siblings to cope with challenges because of the caregiving of their brother/sister with disabilities. Results emphasized the protective role played by these two factors: the social support (40) and the perceived benefits of parentification (55, 56) could act as a buffer preserving the siblings from stressful situations related to the responsibility because of the caregiving of a brother/sister with disabilities. In addition, the perception of benefits because of the parentification could lead the siblings to experience a positive state of mind regarding the caregiving, which, in turn, positively impacts the quality of the relationship (38). Finally, regarding the gender of the siblings and the brother/sister with disabilities as well as the five types of disabilities, in the current study, no significant paths were found. In this vein, further studies were required in order to evaluate this topic.

## 5. Conclusion, Limitations, and implications

Overall, the results of the tested serial mediation model suggested the need to work on the whole family system (both parents, sibling without disability, and brother/sister with disability) simultaneously in order to break the vicious circle through the strengthening of protective factors. This system approach may increase the knowledge regarding the siblings’ needs, enhancing their own personal resources and developing coping strategies and resilience abilities in each family member. It was worth noting that the needs of siblings of people with disabilities could be age-specific; in other words, future intervention programs could take account and promote the achievement of the siblings’ age-specific milestones. Regarding the emerging young adulthood considered in the present study, the main milestones were the development and/or the maintenance of intimate relationships, the achievement of a job, and financial stability (43). These milestones could be hindered by duties because of the caregiving of a brother/sister with disabilities, e.g., a medical visit of the brother/sister with disabilities or the need to help parents in home management could make it difficult for the siblings to go out and/or meet new people; again, requesting work permits frequently or refusing transfers to other cities because of the concerns regarding leaving parents alone in managing the brother/sister with disabilities makes the work life of the siblings more challenging. All these difficulties could lead the siblings to experience distress and high life dissatisfaction. Thus, in order to prevent the onset of siblings’ distress from affecting their wellbeing and life satisfaction, future research could examine which risk and protective sources could be involved in other age ranges (e.g., childhood, adolescence, and young adulthood).

The current findings need to be considered in light of some limits. First, the sample consists mainly of women. Future research should include a balanced sample in order to explore the impact of parentification on men. Second, we did not compare siblings of people with disabilities to those of typically developing individuals as a control group. However, in this study, we aimed at exploring exclusively the impact of risk and protective factors on the relationship between sibling-focused parentification and the sibling relationship in order to inform the family-based intervention strategies. Third, the study was cross-sectional, and future research should test our serial mediation model on longitudinal data. Finally, we evaluated the relationship with parents considering both parents; in order to overcome this limitation, future studies could focus on the quality of the relationship with parents considering each one separately. This would allow exploring the positive and/or negative effects brought by each parent on the considered variables in the present study.

Albeit these limits, findings suggest the following clinical implications. First, the results highlighted the pivotal protective role of perceived social support. Professionals who work with families where disabilities occur could engage the siblings without disabilities in social support intervention programs: referring to them, siblings could share their own feelings with people with similar experiences and learn strategies to better manage their role as caregivers. Furthermore, because of the protective role served by the perceived benefits of parentification, these supportive intervention programs could help the siblings in detecting the positive aspects (e.g., more empathy and the right perception of responsibilities) related to growing up with a brother/sister with disabilities.

In conclusion, the intervention programs could promote the siblings’ state of flourishing (47) (i.e., low mental illness symptoms and high wellness). Leveraging on the protective factors tested in the current study, intervention programs could decrease the distress levels of siblings increasing the awareness regarding (i) the strengths of one’s own brother/sister with disabilities, (ii) personal benefits related to the parentification, and (iii) the social support they could receive from family, health services, and significant others. As a cascade effect, the less distress experienced, the more positive the quality of the family relationships.

## 6. Summary

According to parentification theory, when the siblings of people with disabilities assume adult-like duties, this role reversal is known as sibling-focused parentification. It has a significant impact on siblings’ distress levels and the quality of family relationships. Participants (*n* = 605) were adult siblings (age range: 19–26 years) of people with disabilities. Measures of sibling-focused parentification, distress, and quality of family relationships were used. The hypothesized model aims at testing the relationship between sibling-focused parentification and sibling relationship *via* the mediation of the siblings’ distress and the quality of relationship with their parents. Social support and the benefits of parentification as protective factors were considered. Using the mediation model, results showed that the distress and the low quality of the relationship with parents negatively affected the relationship between sibling-focused parentification and sibling relationship. Findings showed that social support and the perceived benefits of parentification decreased the distress of the siblings; the perceived benefits of parentification served also as protective factors for the quality of the relationship with parents. Current findings extend the knowledge regarding the interplay between these psychological factors on the mental health of people whose siblings are people with disabilities and inform family-based intervention programs that should involve the whole family for reducing distress and improving wellbeing.

## Data availability statement

The raw data supporting the conclusions of this article will be made available by the authors, without undue reservation.

## Ethics statement

The studies involving human participants were reviewed and approved by Ethical Committee for Research in Psychology, Department of Human and Social Sciences, University of Salento, Lecce, Italy. The patients/participants provided their written informed e-consent to participate in this study.

## Author contributions

FL conceived and supervised the study. AL collaborated to the study design, carried out the statistical analyses, and wrote the draft. CM collaborated in statistical analyses. All authors contributed to the data collection, read, and approved the final version of the manuscript.
